# Does mentoring new peer reviewers improve review quality? A randomized trial

**DOI:** 10.1186/1472-6920-12-83

**Published:** 2012-08-28

**Authors:** Debra Houry, Steven Green, Michael Callaham

**Affiliations:** 1 Department of Emergency Medicine, Emory University School of Medicine, Atlanta, GA, USA; 2 Department of Emergency Medicine, Loma Linda School of Medicine, Los Angeles, CA, USA; 3 Department of Emergency Medicine, University of California, San Francisco, CA, USA

**Keywords:** Mentoring, Peer review, Scientific publication, Critical analysis, Journal peer reviewer

## Abstract

**Background:**

Prior efforts to train medical journal peer reviewers have not improved subsequent review quality, although such interventions were general and brief. We hypothesized that a manuscript-specific and more extended intervention pairing new reviewers with high-quality senior reviewers as mentors would improve subsequent review quality.

**Methods:**

Over a four-year period we randomly assigned all new reviewers for *Annals of Emergency Medicine* to receive our standard written informational materials alone, or these materials plus a new mentoring intervention. For this program we paired new reviewers with a high-quality senior reviewer for each of their first three manuscript reviews, and asked mentees to discuss their review with their mentor by email or phone. We then compared the quality of subsequent reviews between the control and intervention groups, using linear mixed effects models of the slopes of review quality scores over time.

**Results:**

We studied 490 manuscript reviews, with similar baseline characteristics between the 24 mentees who completed the trial and the 22 control reviewers. Mean quality scores for the first 3 reviews on our 1 to 5 point scale were similar between control and mentee groups (3.4 versus 3.5), as were slopes of change of review scores (-0.229 versus -0.549) and all other secondary measures of reviewer performance.

**Conclusions:**

A structured training intervention of pairing newly recruited medical journal peer reviewers with senior reviewer mentors did not improve the quality of their subsequent reviews.

## Background

Scientific journals have been regularly subjecting their submitted manuscripts to peer review for over two centuries, and during this period the practice has not changed appreciably [1]. Peer reviewers – most volunteers – are of variable quality. An ongoing challenge for most journals is the recruitment and retention of individuals who can capably inform editors on study strengths, weaknesses, and who can craft accurate feedback to authors on how to improve their manuscripts. Contrary to many editor’s assumptions, the quality of peer reviewers cannot be predicted by their academic rank, formal research training, grant funding, or other traditional markers of academic achievement [2,3].

Since there is no apparent way to preferentially recruit superior reviewers, an alternative recourse is for a journal to attempt to improve the skills of new or existing peer reviewers through training. We have previously studied the impact of providing each reviewer brief written feedback and the editor’s quality score of their reviews. Unfortunately, this intervention did not improve subsequent review performance [4]. Similarly, we implemented structured training workshops that, although popular with attendees, did not lead to better reviews [5,6]. Another journal implemented a self-instructional training package for reviewers that did enhance manuscript error identification; however the impact was marginal and it disappeared altogether after six months [7].

The above interventions were brief, not tailored to each specific review, or both – factors that may account for their failure. In a survey of reviewers at 41 nursing journals, most responded that it took one to five reviews before they were comfortable with the peer review process, with many reporting benefit from specific editor feedback [8]. At our journal 43% of reviewers expressed the desire for more editor feedback [9]. If focused training that is more than brief and tailored to the specific review is indeed effective, then it could improve the quality of peer review and thus ultimately enhance the quality of science.

We hypothesized that we could improve review quality through a mentoring program more extensive and prolonged than previous attempts (but still not dramatically increasing editor workload), in which we paired new reviewers with interested senior reviewers of established high quality.

## Methods

### Study design and setting

We conducted this randomized controlled trial from April 2006 to October 2010 at *Annals of Emergency Medicine*, the leading journal of emergency medicine with 28,000 subscribers and approximately 1,600 submissions per year, 50 editors who make decisions on manuscript acceptance, and 1,000 registered reviewers. The Emory University Institutional Review Board approved the study.

### Journal standard practice

Throughout the study period we continued our standard practice of identifying new reviewer candidates either through self-nomination or editor recommendation, a process that usually adds 10 to 20 reviewers per year to our pool. To be approved for this role, each candidate must have already published at least two first-author peer-reviewed publications. We provided each new reviewer a packet of information materials and encouraged them to complete an online peer review training module developed by our journal [10]. All reviewers are informed once a year that *Annals* may use performance data to assess journal quality and for research purposes, but that all results will be anonymously reported. The same information is included on the invitation email for every review; the reviewer can decline to have their data used for these purposes if they wish. None in this study did so.

Our editors select reviewers as they see fit for any given manuscript, based upon personal knowledge or topic classification matches identified using our editorial management software. All peer review at our journal is blinded; however after a decision is made on each manuscript we routinely provide the reviewer blinded copies of all the comments of other reviewers on that paper, as well as a copy of the editor’s decision letter to the authors.

All reviews at *Annals* are routinely rated by editors for quality on a previously reported 5-point scale that has demonstrated moderate reliability [11] and is comparable to the scale validated by van Rooyen [12]. The gold standard for this score is the usefulness of the review to the editor (and authors), consisting of 6 essential specific components described in detail elsewhere [11,13].

### Selection of participants

We enrolled consecutive individuals newly added to our reviewer ranks during the study period. There were no exclusion criteria. From their submitted curriculum vitae we appraised their prior experience and publications (Table 1).

**Table 1:** Baseline characteristics of study subjects

**Characteristic**	**Control Group (n = 22)**	**Mentored Group (n = 24)**
*Prior peer review experience*		
Any prior peer review	11	14
Prior peer review for 3 or more other journals	8	4
Prior peer review experience with a journal of higher impact than *Annals*	7	5
*Prior authorship experience*		
Median number of first-author publications in a peer-reviewed journal (range)	3 (0 to 15)	4 (1 to 20)
Median number of first-author publications in a peer-reviewed journal of higher impact than *Annals* (range)	0 (0 to 3)	1 (0 to 3)
*Self-reported average usefulness score* of various experiences to their peer review skills (Likert scale 1 low, 5 high), with response rates shown		
Previous peer review experience at another journal	3.5 (n = 14)	4.2 (n = 17)
Formal training course in peer review	3.1 (n = 8)	3.6 (n = 14)
Formal training in critical appraisal	4.0 (n = 10)	4.0 (n = 17)
Mentorship at *Annals* from editors or other reviewers	3.3 (n = 7)	3.5 (n = 20)
Other mentoring	3.4 (n = 9)	3.4 (n = 11)
Instructional articles or media on peer review	3.2 (n = 11)	2.7 (n = 16)
*Number of experiences* of any category of training or mentoring (excluding at a previous journal) listed above (95% CI) p = .003	2.6 (2.1 to 3.2) (n = 17)	3.9 (3.4 to 4.4) (n = 20)

Our mentors were senior journal reviewers who responded affirmatively to an email request for volunteers to mentor new reviewers. To be a senior reviewer, they had to make the “top 50” reviewer list for at least two of the past four years. This performance list ranks them by a formula that includes their overall timeliness, quality scores, and review volume. These mentors were thus an elite group within the top 5% of our reviewers overall.

### Intervention

We used computer-generated randomization to assign new reviewers to either the intervention or control group. A senior editor emailed those in the intervention group and invited them to participate in a new mentoring program. Once agreed, they were provided detailed instructions on how to proceed. Those in the control group received no initial study-specific contacts and no intervention beyond standard journal practices outlined above. Over the course of the study editors invited reviewers in their standard fashion, without knowledge of which new reviewers were assigned to the mentorship or control groups.

When mentors were invited and subsequently agreed to review a given manuscript, the managing editor of the journal then perused the list of new reviewers in the intervention group, and selected one with similar topic expertise (if available). This paired mentee was then assigned the same manuscript to review.

By this method we assembled paired mentees and mentors reviewing the same manuscript. Mentees were asked to discuss their review with their mentor by email or telephone. Mentors were asked to give feedback about how well the mentee addressed the key elements of a good review, what they might have done differently in the review, and how they would rate their own review on *Annals’* 1 to 5 point quality scale. The content and amount of communication were left to the mentor and mentee.

### Outcome measurements

Our main outcome measures were the mean review quality rating score for each reviewer, and the slope in change (improvement or deterioration) in editor-assigned reviewer quality ratings from their first review until the end date of the study. These were calculated from review scores recorded contemporaneously within our editorial management software.

Our secondary outcomes were mentee satisfaction and perspectives on the program. To collect this information, we surveyed each mentee after three mentored reviews were completed. We asked mentees how many total contacts they had had with their mentor(s), whether they used email or telephone, and their opinions regarding the program.

At the conclusion of the study period we sent an identical survey to both intervention and control groups asking them to rate on a 1–5 scale how useful the following features contributed to their peer review experience: (1) previous peer review experience at other journals, (2) prior formal training course(s) in manuscript peer review, (3) prior formal training in critical appraisal, (4) unstructured mentoring experience at *Annals*, or (5) instructional articles or other media on how to review a manuscript.

### Data analysis

We analyzed changes in review quality scores over time (the slope of quality score trends) using linear mixed effect models [14,15] with Stata 10 (StataCorp, College Station, TX), in accordance with methodology detailed elsewhere [13]. The resulting model controls for within-reviewer and between-reviewer trends, as well as between-editor and within-editor trends, and calculates the slope of change in an individual reviewer’s scores over time.

We were unable to perform a sample size calculation given the unreliability of the necessary baseline assumptions. Instead, we chose to enroll a 4-year consecutive sample.

## Results

**Figure 1:**
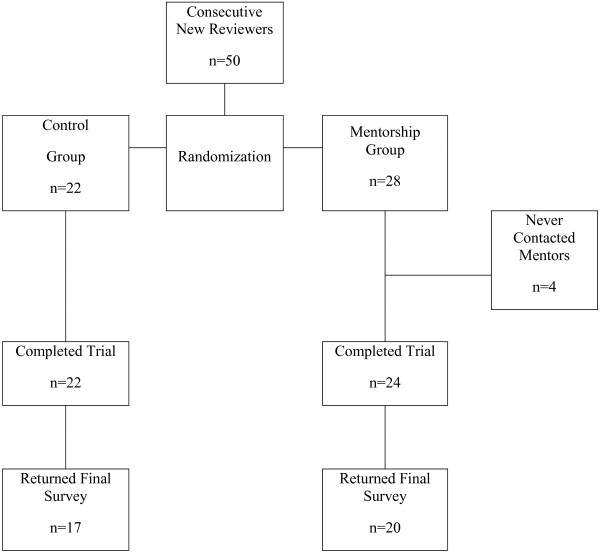
Participant flow.

Participant flow is shown in Figure 1. Four subjects randomized to the mentorship group never made contact of any kind with their mentors, and thus did not receive the intended intervention. We therefore present below the per-protocol analysis excluding these four subjects; however we also performed a corresponding intention-to-treat analysis that yielded essentially identical results (data not shown). 37 reviewers returned the survey of their experience (17 controls and 20 mentees); of these all had had at least one category of prior training (see Table 1).

The baseline characteristics between study groups were mostly similar (Table 1). These included prior experience with peer review at other journals (83% of both groups), authorship experience, and exposure to potentially helpful experiences such as training courses in peer review or critical appraisal.

During the study period the participants were invited to perform 912 reviews, and accepted and completed 490 reviews. Their performance is detailed in Table 2. The mean quality scores over the first 3 reviews on our 1 to 5 point scale were similar between groups: 3.4 (95% CI 3.1 to 3.9) for controls and 3.5 (3.2 to 3.9) for mentees (Table 2). (Our score defines 3 as “acceptable”, 4 as “good”, and 5 as “exceptional, hard to improve”.) This size of effect was 0.1, with 95% CI of −0.4 to 0.6. For our primary outcome, the slope of quality score change was also similar: -0.229 (95% CI −0.644 to −0.185) for controls and −0.549 (−0.831 to −0.267) for mentees. The mentees reported having contacted their mentor an average 2.6 times during the first 3 reviews (95% CI 2.0 to 3.2).

**Table 2:** Performance of new reviewers by group

**Variable**	**Control (n = 22)**	**Mentored (n = 24)**
Mean number of invitations to review (95%CI)	15 (5 to 24)	23 (14 to 32)
Mean number of reviews declined (95%CI)	2 (0 to 4)	5 (3 to 6)
Percent of invitations declined (95%CI)	13% (5% to 22%)	23% (15% to 32%)
Mean number of reviews completed (95%CI)	10 (6 to 14)	10 (6 to 14)
Mean review score all reviews (95%CI)	3.4 (3.1 to 3.8)	3.5 (3.2 to 3.9)
Mean review score for the first 3 reviews (95%CI)	3.5 (3.1 to 3.9)	3.5 (3.2 to 3.9)
Mean slope of change of review scores (95%CI)	-0.23 (-0.66 to -0.18)	-0.55 (-0.83 to -0.27)

## Discussion

Despite the fact that peer reviewers play a major role in selecting what science is published (and thereby “endorsed”), little is understood on how to select the best reviewers or improve the skills of existing ones. Past studies of training and mentoring, including a controlled trial of performance feedback to new reviewers, have shown no objective benefit [4].

We hypothesized that perhaps these failures were due to an insufficiently focused and detailed mentoring process, which has been deemed necessary in previous studies of teaching complex writing skills [16]. We therefore attempted to develop a more individualized and detailed approach that would still not represent too great a burden on the journal or the editors. All reviewers newly added to *Annals of Emergency Medicine* during a four-year period were randomly assigned to a control group or a mentoring group. Both groups were assigned papers in our usual fashion based on their availability and topic expertise. The control group performed their review and was informed of the editor’s final decision, as well as being given access to the full reviews of the same manuscript by other reviewers after the decision was made. The mentee group was treated similarly except that they were advised at the start of the study of the availability of a specific named mentor volunteer and encouraged to discuss papers individually with that reviewer either by phone or email. The enrolled reviewers were surveyed for relevant experience and training in peer review and critical analysis, based on a review of their curriculum vitae and a questionnaire. We found no differences between the two groups from this review, with the exception that the control group had had a lesser total number of formal training experiences than the mentored group (Table 1).

Despite this one-on-one mentoring in the intervention group, there were no differences in mean reviewer quality scores between groups, using a validated scale routinely used at this journal for over 20 years [11]. One might expect that the mentoring intervention would have greatest impact on the first three reviews performed, as compared to all reviews performed during the course of the study. However, we found no difference when conducting this sub-analysis. We also examined the performance trend of all reviewers (change in quality scores over time), using a mixed linear effects model reported in detail elsewhere, which corrects for editor and reviewer variables [13]. This method of performance measurement also showed no difference between the groups.

We asked the study participants for free text comments about the mentoring experiences and approximately half provided comments. The majority of comments were neutral (including those who got mentoring elsewhere or felt what they learned was mostly specific journal format or style), 3 were positive, and only 3 very positive.

Since the majority of reviewers at the journal *Medical Education* wanted formal training in reviewing and 80% would have liked to seek a colleague’s opinion [17], we thought that assigning a senior reviewer with a junior reviewer with similar expertise topics might serve this purpose. However this was apparently not the case. Previous studies have reported that written feedback to reviewers, workshops, and self-taught training packages did not result in lasting improvements in peer review (4–7).

For those who might think this lack of efficacy is aberrant or unique to our journal environment, similar results have been reported in regards to teaching physicians critical appraisal skills in other settings. A Cochrane review cited found only one randomized trial on teaching critical appraisal skills rigorous enough and stated that conclusions about the effects of teaching critical appraisal are debatable [18]. Another educational trial which randomized practitioners to half-day critical appraisal skills training workshop or wait list control found that those who took their course had a greater overall knowledge score, but no differences in overall attitude towards evidence, perceived confidence, and other areas of critical appraisal skills ability (methodology or generalizability) [19]. Finally, a systematic review of journal clubs reported that studies showed an improvement in knowledge of clinical epidemiology and biostatistics, but no improvement in critical appraisal skills [20].

Our study was limited by several factors. The sample size was small, but it included all new reviewers over a four-year period, and the confidence intervals on our mean scores were not wide, limiting the potential for type 2 error. (This size of effect was 0.1, with 95% CI of −0.4 to 0.6). This study was also conducted only at a single specialty journal. However, given other studies demonstrating similarity in characteristics between our reviewers and those at other journals and specialties [2,4-6,11,13,21], it seems unlikely that this intervention would yield significantly different results elsewhere. Our mentors were not provided specific training in mentoring techniques, although most worked in academic settings where mentoring skills would be high. They had however a proven track record of high quality reviews over a long period of time, and an expressed willingness to mentor others. Mentors and mentees were encouraged to communicate by email or phone, but were not given more explicit or rigid guidelines, since in all regards we were aiming for an intervention that was logistically feasible and likely to be implemented by journals. As well, the actual mentoring and communication was not observed or evaluated by any outside party, so we cannot comment on its consistency. It is possible that the results of this study might have been different had the mentors all had formal training specific to this goal and/or the communication between mentor and mentee had been more standardized, more frequent, or mandated for a longer period of time. We did not implement these requirements because we felt that all these changes would limit compliance and would make it much less likely that a typical journal would invest the energy in implementing this technique.

The absence of observed efficacy in our study might be theorized to have occurred because all of our new reviewers perform at a relatively high level of function and thus the potential margin for improvement is too small to be significant. Our routine processes may better prepare our reviewers for their tasks than at some other journals. Upon recruitment we refer all new reviewers to our training module [10], (although we do not enforce its usage), and upon the completion of each review we provide them access to comments from the other reviewers and the editor. Additionally, many of our reviewers, although new to our journal, had prior experience reviewing at other journals, or had taken formal training, or both, and all had had at least one form of prior training or mentorship (Table 1).

However, this explanation seems less likely because this sample included all reviewers, including those self-referred, and no screening was performed (or possible) to select higher quality reviewers in advance [2]. The study cohort had an average quality score not significantly different than the 3.61 (95% CI 3.57 – 3.61) of the larger and longer-term reviewer pool [15]. Similarly, their slope of change over time was similar to the slope of −0.04 (−0.039 to −0.042) for the larger pool [13]. Since on our scale these scores are between an “acceptable” and “commendable”, there is plenty of room left for improvement. Yet more evidence against the explanation that our reviewers were atypically trained and adept, the mentoring group had a significantly greater number of training experiences than the control group (Table 2) but the better performance expected did not materialize.

This study adds to the list of those that have not found a successful formula for improving reviewer performance. The reasons for this are as yet unproven, but a major one may be that teaching and improving writing skills is a very complex task which can only be accomplished by very extensive mentoring, ideally provided very promptly, with a rapid opportunity for the learner to absorb feedback, practice and improve their performance. None of these characteristics is present in the peer review process of most journals; feedback is minimal and provided long after the reviewer’s critical thinking is completed. The feedback needed to improve high level analytic and writing skills is particularly detailed and time consuming for both advisor, and advisee, far beyond the resources of even the largest journals to provide [16,22]. This is especially true since most participants in the process are unpaid volunteers, and internal quality assurance programs at journals are uncommon.

## Conclusions

A simple system of pairing newly recruited peer reviewers with volunteer reviewer mentors and encouraging limited but direct discussion of the papers did not result in higher review scores. There are no proven measures for screening or improving the skills of peer reviewers, who are the gatekeepers of published science. This makes careful and permanent monitoring of reviewer performance all the more important, a practice still not followed by many journals.

## Competing interests

The authors declare that they have no competing interests.

## Authors’ contributions

MC, DH, and SG participated in the study design. DH, SG, and MC participated in data collection. MC and SG participated in data analysis. All authors participated in drafting the manuscript, interpreting the data, and approving the final manuscript.

## Pre-publication history

The pre-publication history for this paper can be accessed here:

http://www.biomedcentral.com/1472-6920/12/83/prepub
